# Alterations in Mitochondrial Quality Control in Alzheimer’s Disease

**DOI:** 10.3389/fncel.2016.00024

**Published:** 2016-02-09

**Authors:** Qian Cai, Prasad Tammineni

**Affiliations:** Department of Cell Biology and Neuroscience, Rutgers, The State University of New JerseyPiscataway, NJ, USA

**Keywords:** mitochondrial quality control, mitochondrial dynamics, mitophagy, mitochondrial transport, mitophagosome, Alzheimer’s disease, axonal transport

## Abstract

Mitochondrial dysfunction is one of the earliest and most prominent features in the brains of Alzheimer’s disease (AD) patients. Recent studies suggest that mitochondrial dysfunction plays a pivotal role in the pathogenesis of AD. Neurons are metabolically active cells, causing them to be particularly dependent on mitochondrial function for survival and maintenance. As highly dynamic organelles, mitochondria are characterized by a balance of fusion and fission, transport, and mitophagy, all of which are essential for maintaining mitochondrial integrity and function. Mitochondrial dynamics and mitophagy can therefore be identified as key pathways in mitochondrial quality control. Tremendous progress has been made in studying changes in these key aspects of mitochondrial biology in the vulnerable neurons of AD brains and mouse models, and the potential underlying mechanisms of such changes. This review highlights recent findings on alterations in the mitochondrial dynamics and mitophagy in AD and discusses how these abnormalities impact mitochondrial quality control and thus contribute to mitochondrial dysfunction in AD.

## Introduction

Mitochondria are organelles essential for neuronal function and survival (Nicholls and Budd, 2000; Sheng and Cai, 2012; Mishra and Chan, 2014). Their key role is ATP production, which is vital for maintaining neuronal integrity and function (Verstreken et al., 2005; Sun et al., 2013; Rangaraju et al., 2014). Mitochondria also play a critical role in buffering intracellular Ca^2+^ levels by taking up and releasing Ca^2+^. At synaptic terminals, mitochondria take up excess intracellular Ca^2+^ and release Ca^2+^ to prolong residual levels, maintaining Ca^2+^ homeostasis (Tang and Zucker, 1997). Through this mechanism, synaptic mitochondria are thought to be involved in the regulation of neurotransmission (Billups and Forsythe, 2002; David and Barrett, 2003) or certain types of short-term synaptic plasticity (Levy et al., 2003; Kang et al., 2008). Dysfunctional mitochondria not only produce energy and buffer Ca^2+^ less efficiently, but also release harmful reactive oxygen species (ROS; Court and Coleman, 2012; Sheng and Cai, 2012). As a result, mitochondrial oxidative stress triggers leakage of mitochondrial intermembranous contents, such as cytochrome c, into the cytosol, causing caspase activation, DNA damage, and apoptosis (Mishra and Chan, 2014). The progressive accumulation of these damaged mitochondria in axons and synapses over the lifetime of neurons is thought to contribute to the pathogenesis of Alzheimer’s disease (AD; Du et al., 2012; Reddy, 2013).

The half-life of neuronal mitochondria is estimated to be ~30 days (Gross et al., 1969; Menzies and Gold, 1971). Throughout a neuron’s lifetime, aged and damaged mitochondria undergo dynamic recycling via fusion and fission or elimination via mitophagy, a cargo-selective autophagy that degrades mitochondria within lysosomes after their transport back to the soma. These dynamic processes of mitochondrial fusion, fission, transport, and turnover constitute the elaborate system of mitochondrial quality control, which regulates mitochondrial function by enabling recruitment of healthy mitochondria to subcellular compartments with high demands for ATP, such as synaptic terminals or axonal branches (Bogan and Cabot, 1991); content exchange between mitochondria; mitochondrial shape control; and mitochondrial turnover via mitophagy (Chen and Chan, 2009; Sheng and Cai, 2012). Disruptions in any of these processes lead to mitochondrial pathology, cellular dysfunction, and neurological defects (Chen and Chan, 2009).

Mitochondrial dysfunction is a significant concern in the nervous system of aging and it has been associated with major neurodegenerative disorders including the devastating AD that now affects 50% of individuals over 85 years old (Chen and Chan, 2009; Sheng and Cai, 2012). Mitochondrial deficiency has been suggested to be a hallmark of AD as the patients display early metabolic changes prior to the emergence of any histopathological or clinical abnormalities (Gibson and Shi, 2010). Mitochondrial pathology is also thought to contribute to synaptic deficits, an early pathophysiological feature of AD (Du et al., 2012; Reddy, 2013). Other prominent mitochondria-related anomalies in AD include the accumulation of damaged mitochondria in both familial and sporadic forms of the disease (Swerdlow et al., 2010), as well as changes in mitochondrial structure, dynamics, and motility in vulnerable neurons of affected brain regions (Baloyannis, 2006; Chen and Chan, 2009; Sheng and Cai, 2012). Here, we provide an overview of the underlying mechanisms regulating mitochondrial dynamics and transport and discuss how abnormalities in these mechanisms compromise mitochondrial quality control, thus contributing to mitochondrial dysfunction in AD.

### Mitochondrial Fission

**Figure 1:**
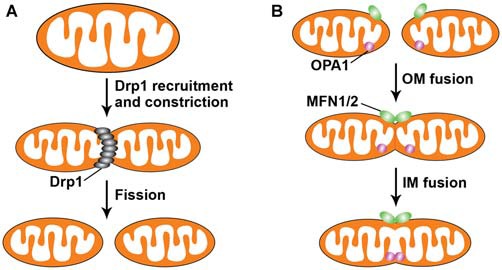
**Mitochondrial fission and fusion.** Mitochondria are dynamic organelles that undergo continuous fusion and fission events to intermix their lipids and contents. **(A)** Dynamin-related protein 1 (DRP1) regulates mitochondrial fission, which consists of two steps: first, DRP1 is recruited from the cytosol to the mitochondrial outer membrane (OM); second, its assemblage on the mitochondrial surface results in constriction of the mitochondria, leading to the separation of one mitochondrion into two entities. **(B)** Mitofusins 1 and 2 (MFN1/2) at the OM and optic atrophy 1 (OPA1) at the inner membrane (IM) orchestrate mitochondrial fusion, which involves MFN1/2-mediated OM fusion of two mitochondria, followed by OPA1-directed IM fusion. Mitochondrial fusion leads to elongated and highly interconnected mitochondria.

Mitochondria constantly change their shape through continuous fusion and fission events (Figure 1). Mitochondrial fission in mammals requires Drp1, a dynamin-like protein (Detmer and Chan, 2007; Reddy et al., 2011; Kandimalla and Hemachandra Reddy, 2015). Drp1 is a cytosolic protein recruited to mitochondria during fission, forming a ring-like higher order structure that physically pinches the mitochondria into two daughter mitochondria. Drp1-mediated mitochondrial fission in yeast requires an additional mitochondrial outer membrane (OM) protein, Fis1 (Okamoto and Shaw, 2005). Knocking down Fis1 in mammalian cells was also found to block mitochondrial fission without affecting Drp1 localization to mitochondria (Lee et al., 2004). Posttranslational modifications of Drp1 appear to be important for regulating Drp1 activity. As a proposed substrate of Sumo 1, Sumo 1-dependent sumoylation protects Drp1 from degradation, thus promoting mitochondrial fission (Harder et al., 2004). Loss of Parkin or PINK1 function in human SH-SY5Y cells also resulted in increased Drp1-dependent mitochondrial fragmentation (Lutz et al., 2009). Consistently, a study showed that Parkin ubiquitinates Drp1 for its degradation within the proteasome system (Wang et al., 2011a). However, it was also found that loss of PINK1 in *Drosophila* impairs mitochondrial fission (Liu et al., 2011). Another mitochondrial E3 ubiquitin ligase known to regulate Drp1-mediated fission is MARCH-V, which mediates ubiquitination of Drp1 (Nakamura et al., 2006; Karbowski et al., 2007). Phosphorylation of Drp1 at different sites, which functions variably to either promote or inhibit its activity, is another mechanism regulating mitochondrial morphology (Chang and Blackstone, 2007; Cribbs and Strack, 2007; Taguchi et al., 2007). Although no inherited diseases caused by mutations of Drp1 have been reported, Drp1 is necessary for embryonic development of the mouse brain and for synapse formation in cultured neurons (Ishihara et al., 2009). In addition, Drp1 maintains the proper distribution of mitochondria near *Drosophila* neuromuscular junctions (Verstreken et al., 2005). It was shown that defects in Drp1-mediated mitochondrial fission result in the accumulation of mitochondria in the cell body and reduced dendritic mitochondrial content (Li et al., 2004).

### Mitochondrial Fusion

Mitochondrial fusion in mammals is mainly mediated by three proteins: Mfn1, Mfn2 and OPA1 (Mishra and Chan, 2014; Roy et al., 2015). Mfn1 and Mfn2 are required for OM fusion, whereas OPA1 is involved in inner membrane (IM) fusion (Meeusen et al., 2006; Song et al., 2009). Recent studies propose that long forms of OPA1 is sufficient for mediating efficient fusion and that short OPA1 forms may act independently to promote mitochondrial fission (Ishihara et al., 2006; Anand et al., 2014). OPA1 proteolysis itself has been reported to be a profusion event that operates at the time of fusion (Mishra et al., 2014). Mutations of each of these genes have been linked to neurological disorders. Mutations in Mfn2 cause Charcot-Marie-Tooth type 2A (CMT2A), a peripheral neuropathy affecting sensory and motor neurons (Zuchner et al., 2004), while mutations in OPA1 are the primary cause of autosomal dominant optic atrophy (DOA), a degeneration of retinal ganglia cells that results in atrophy of the optic nerve (Alexander et al., 2000; Delettre et al., 2000). Deletion of each of these genes in mice leads to mitochondrial dysfunction and embryonic lethality (Chen et al., 2003; Alavi et al., 2007; Davies et al., 2007). In addition to regulating mitochondrial shape, mitochondrial fusion affects the distribution and transport of mitochondria in neurons. Mitochondria in Mfn2 conditional knockout mice are fragmented and absent from distal neurites, impeding dendritic outgrowth, formation of axonal projections, and neuronal survival (Chen et al., 2007; Lee et al., 2012; Pham et al., 2012). Moreover, motor neurons derived from transgenic mice expressing mutant Mfn2 showed improper mitochondrial distribution characterized by tight clusters of mitochondria within axons (Detmer et al., 2008). Recent studies suggest that mitochondrial transport is regulated through fusion machinery by showing that Mfn1 and Mfn2 interact with Miro and Milton, members of the adaptor complex linking mitochondria to kinesin motors. Loss of Mfn2 or expression of Mfn2 disease mutants in neurons was shown to affect axonal mitochondrial fusion and transport and produce classic features of segmental axonal degeneration (Misko et al., 2010, 2012). A study in living zebrafish revealed the contribution of mitofusins, and thus fusion, to mitochondrial transport, which is essential for maintaining motor function (Chapman et al., 2013). Moreover, the motor neurons obtained from differentiation of CMT patient-derived iPS (induced pluripotent stem cells) presented abnormalities in mitochondrial trafficking and abnormal electrophysiological properties (Saporta et al., 2015). Thus, these studies provide evidence that a clear link exists between mitochondrial fusion and transport in neurons.

Mitochondrial fission and fusion ensure optimal functioning of mitochondria. Mitochondrial fusion allows the exchange of mitochondrial metabolites, mitochondrial DNA (mtDNA), and oxidative phosphorylation components within a mitochondrial network. While damaged mitochondria can be repaired through fusion with healthy mitochondria for mixture of contents, mitochondrial elongation upon nutrient deprivation maximizes energy production and promotes cell survival by preventing autophagy-mediated degradation of mitochondria (Gomes et al., 2011; Rambold et al., 2011). On the other hand, mitochondrial fission enables the segregation of mitochondria that become severely and irreversibly damaged or are fusion-incompetent, thereby leading to their subsequent elimination via mitophagy (Twig et al., 2008). Defects in either mitochondrial fusion or fission also lead to impaired mitochondrial motility and distribution in neurons, resulting in reduced mitochondrial content in distal neurites. Together, mitochondrial fusion and fission are required for the maintenance of mitochondrial shape, integrity, and distribution.

## Mitochondrial Transport in Neurons

Altered mitochondrial transport is one of the pathogenic changes in major neurodegenerative diseases (Sheng and Cai, 2012). In mature neurons, ~20–30% of axonal mitochondria are motile while the remaining two thirds are stationary (Cai et al., 2005; Kang et al., 2008). Long-distance transport of mitochondria along microtubules (MTs) between the soma and distal processes or synapses is dependent on MT-based motor proteins, which drive their cargoes via mechanisms requiring ATP hydrolysis (Martin et al., 1999; Hirokawa et al., 2010). MTs are uniformly organized in axons, with the plus end pointed away from the soma and the minus end directed toward the soma (Hirokawa et al., 2010). Thus, kinesin motors, the plus-end driven motors, participate in anterograde axonal transport from the soma to nerve terminals, whereas the minus-end directed dynein motors mediate retrograde transport back to the soma. Efficient regulation of mitochondrial transport is essential for recruiting and redistributing mitochondria to specific domains with high-energy demands, such as synaptic terminals. Mitochondrial transport also plays a critical role in removing aged and damaged mitochondria, and replenishing them with healthy ones at distal regions of neurons.

The kinesin-1 family (KIF5) is the major motor that drives mitochondrial transport (Hurd and Saxton, 1996; Tanaka et al., 1998; Górska-Andrzejak et al., 2003; Cai et al., 2005; Pilling et al., 2006). KIF5 heavy chain (KHC) has a motor domain with ATPase activity located at the N terminus and a C-terminal tail domain required for binding its cargo. Adaptor proteins such as *Drosophila* protein Milton enable KIF5 motors to attach to mitochondria. Milton acts as a KIF5 motor adaptor by binding to both the KIF5 C-terminal tail domain and the mitochondrial OM receptor Miro (Stowers et al., 2002; Glater et al., 2006). Consistently, Milton mutation in *Drosophila* reduces mitochondrial trafficking into synapses. Two Milton orthologues, Trak1 and Trak2, are found in mammals (Smith et al., 2006; MacAskill et al., 2009; Koutsopoulos et al., 2010). Trak2 overexpression in cultured hippocampal neurons robustly enhances axonal mitochondrial motility (Chen and Sheng, 2013). In contrast, loss of Trak1 or expression of its mutants leads to a reduction in mitochondrial transport along axons (Brickley and Stephenson, 2011). A recent study showed that mammalian Trak1 and Trak2 each contain one N-terminal KIF5B binding site and two dynein/dynactin-binding sites, one located at the N-terminus and the other at the C-terminus (van Spronsen et al., 2013). This suggests that the Trak proteins can mediate both KIF5- and dynein-driven bi-directional mitochondrial transport.

**Figure 2:**
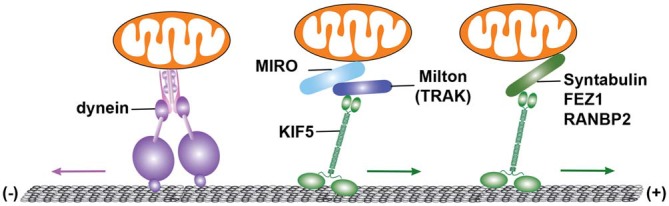
**Microtubule-dependent motor-driven mitochondrial transport.** Cytoplasmic dynein motors and kinesin-1 family (KIF5) of the KIF5 mediate mitochondrial transport. Dynein motors, the minus-end driven motors, carry out retrograde transport of mitochondria toward the soma of neurons. By contrast, KIF5 selectively moves mitochondria toward the plus-end of microtubules (MTs), and participates in anterograde transport from the soma to distal axons and synaptic terminals. Mitochondrial transport driven by KIF5 requires the mitochondrial rho (Miro)-Milton (or Miro-Trak) motor-adaptor complex. MIiro or MIRO is a mitochondrial outer membrane (OM) protein of the Rho GTPase family. In *Drosophila* melanogaster, Milton recruits KIF5 to mitochondria by binding to Miro. In a similar way, Trak1 and Trak2 (mammalian Milton orthologues) can bind to Miro1 and Miro2 (mammalian orthologues of Miro). The Miro1-Trak2 complex is an important regulator of mitochondrial transport in hippocampal neurons. KIF5 also associates with mitochondria and mediates mitochondrial anterograde transport via syntabulin, a KIF5 adaptor that binds to mitochondria via its carboxy-terminal transmembrane domain. Fasciculation and elongation protein zeta-1 (FEZ1), as well as RAN-binding protein 2 (RANBP2), are additional kinesin adaptors that may contribute to mitochondrial transport. Figure is modified from Sheng (2014).

Miro or MIRO, the mitochondrial OM receptor that binds to Trak/Milton, is a Rho-GTPase that consists of two Ca^2+^-binding EF-hand motifs and two GTPase domains (Frederick et al., 2004; Fransson et al., 2006). In *Drosophila*, mutation of the miro gene impairs mitochondrial anterograde transport and depletes the supply of mitochondria in distal synaptic terminals (Guo et al., 2005). Mammalian Miro has two isoforms, Miro1 and Miro2. The Miro1-Trak2 adaptor complex regulates mitochondrial transport in hippocampal neurons (MacAskill et al., 2009). A recent study showed that elevated Miro1 expression increased mitochondrial transport, likely by recruiting more Trak2 and KIF5 motors to mitochondria (Chen and Sheng, 2013). KIF5, Milton (Trak), and Miro are thus assembled into the transport machinery that drives mitochondrial anterograde transport. Syntabulin similarly serves as a KIF5 adaptor for neuronal mitochondria (Cai et al., 2005). Loss of syntabulin or interruption of KIF5-syntabulin coupling reduces mitochondrial anterograde transport in axons. In addition, a number of other proteins have been suggested as KIF5 motor adaptor candidates for mitochondrial transport, including FEZ1 (fasciculation and elongation protein zeta-1) and RAN-binding protein 2 (RANBP2; Cho et al., 2007; Fujita et al., 2007; Figure 2).

Cytoplasmic dynein motors are composed of multiple subunits including heavy chains (DHC) that function as the motor domain for force production, and intermediate (DIC), light intermediate (DLIC), and light chains (DLC) that function in cargo attachment and motility regulation. Dynein motors associate with *Drosophila* mitochondria, and mutations of DHC alter both velocity and run length of retrograde transport of axonal mitochondria (Pilling et al., 2006). In addition to its role as a KIF5 motor adaptor, Miro may also serve as an adaptor for dynein motors in *Drosophila* (Guo et al., 2005; Russo et al., 2009). Loss of dMiro impairs both kinesin- and dynein-driven transport, while dMiro overexpression alters mitochondrial transport in both directions. It is likely that regulation of the opposite-direction motor activity is achieved through modulation of the cargo-adaptor proteins. The existence of multiple motor adaptors suggests that complex mechanisms regulate mitochondrial motility in response to various physiological and pathological conditions.

## Mitophagy

Mitophagy, a selective autophagy for the removal of dysfunctional mitochondria, constitutes a key cellular pathway in mitochondrial quality control. It involves sequestering damaged mitochondria into autophagosomes and subsequently degrading them within lysosomes. Recent studies indicate that PINK1/Parkin-mediated mitophagy ensures mitochondrial integrity and function (Clark et al., 2006; Gautier et al., 2008; Narendra et al., 2008). This type of mitophagy is initiated with stable accumulation of PTEN-induced putative kinase protein 1 (PINK1) on the surface of damaged mitochondria, followed by recruitment of Parkin from the cytosol to the mitochondria. Parkin, an E3 ubiquitin ligase, then ubiquitinates a number of mitochondrial OM proteins and activates the ubiquitin-proteasome system (Geisler et al., 2010; Poole et al., 2010; Ziviani et al., 2010; Chan et al., 2011; Yoshii et al., 2011), which works in conjunction with the AAA ATPase p97 to degrade mitochondrial OM proteins (Tanaka et al., 2010; Chan et al., 2011). This triggers engulfment of damaged mitochondria by isolation membranes to form autophagosomes. Loss-of-function mutations of PINK1 and Parkin are associated with autosomal recessive forms of Parkinson’s disease. While the functions of PINK1 and Parkin in mitophagy have been proposed mostly from *in vitro* studies, their involvement in this pathway has recently been supported by proteomic analyses in *Drosophila* under normal physiological conditions (Vincow et al., 2013). The PINK1/Parkin pathway thus promotes both mitophagy *in vivo* and the selective turnover of mitochondrial respiratory chain subunits.

**Figure 3:**
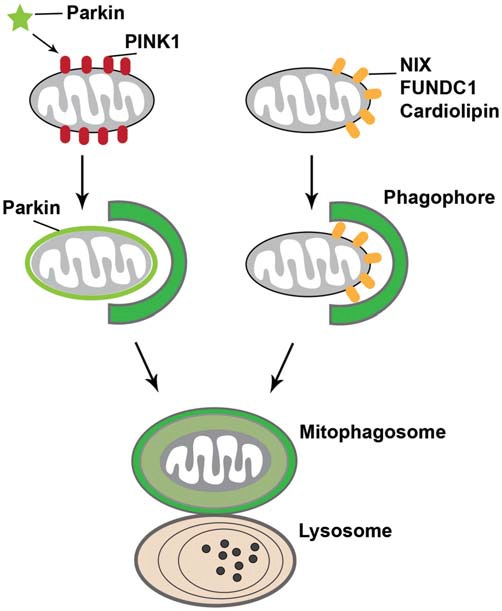
**Mitophagy.** Mitophagy, which is initiated when damaged mitochondria are labeled for their subsequent recruitment into phagophore or isolation membranes, occurs through two mechanisms. First, upon loss of mitochondrial membrane potential, the E3 ubiquitin ligase Parkin is recruited from the cytosol to damaged mitochondria in a PTEN-induced putative kinase protein 1 (PINK1)-dependent manner. Parkin ubiquitinates mitochondrial proteins and causes mitochondria to become engulfed by phagophore or isolation membranes that then fuse with lysosomes. Second, outer mitochondrial membrane proteins, such as NIP3-like protein X (NIX; also known as BNIP3L), FUN14 domain containing 1 (FUNDC1), or cardiolipin externalized from the inner mitochondrial membrane phospholipid upon mitochondrial damage, bind to LC3 on the phagophore or isolation membranes, which mediate the sequestration of damaged mitochondria into mitophagosomes for lysosomal degradation.

Other PINK1/Parkin-independent mitophagy pathways have been identified as well. For instance, a mitochondrial OM protein, the BCL-2 homology 3 (BH3)-containing protein NIP3-like X (NIX, also known as BNIP3L), was shown to play an important role in the elimination of mitochondria in erythrocytes (Sandoval et al., 2008). NIX contains an amino-terminal LC3-interacting region (LIR) that binds to LC3 on isolation membranes (Novak et al., 2010). This enables NIX to act as a selective mitophagy receptor that physically connects the autophagy machinery to the mitochondrial surface in erythroid cells. Another mitochondrial OM protein that has been proposed as a mitophagy receptor is FUN14 domain containing 1 (FUNDC1), which regulates the autophagic degradation of mitochondria in response to hypoxia. FUNDC1 has a LIR required for recruitment of LC3 (Liu et al., 2012a). Under hypoxic conditions, this LIR is dephosphorylated by the mitochondrial phosphatase phosphoglycerate mutase family member 5 (PGAM5), thus increasing its physical association with LC3 and promoting mitophagy (Chen et al., 2014). In addition, a recent study reported that in neuronal cells, cardiolipin—an inner mitochondrial membrane phospholipid—externalizes to the OM upon mitochondrial damage. LC3 has been shown to contain cardiolipin-binding sites, which suggests that externalized cardiolipin acts as an elimination signal for neuronal mitophagy (Chu et al., 2013). Altogether, these observations indicate that specific mitophagy receptors on the mitochondrial OM play an essential role in mitochondrial degradation by recruiting autophagy machinery to mitochondria (Figure 3).

As a highly dynamic process, mitophagy needs to be evaluated using several complementary assays: (1) cellular analysis of depolarization of mitochondrial membrane potential with Parkin translocation, co-localization with autophagy markers (mitophagosome formation), and lysosomal sequestration of defective mitochondria; (2) ultrastructural analysis by electron microscopy based on alterations in mitochondrial structure and morphological features of autophagic vacuoles (AVs) containing mitochondrial profiles; and (3) western blot analysis of increased association of mitophagy machinery with mitochondria and reduced levels of mitochondrial proteins upon mitophagy induction. These assays need to be combined with the application of mitochondrial membrane potential dissipating agents and a flux inhibitor to trap newly formed autophagosomes.

## Mitochondrial Quality Control

**Figure 4:**
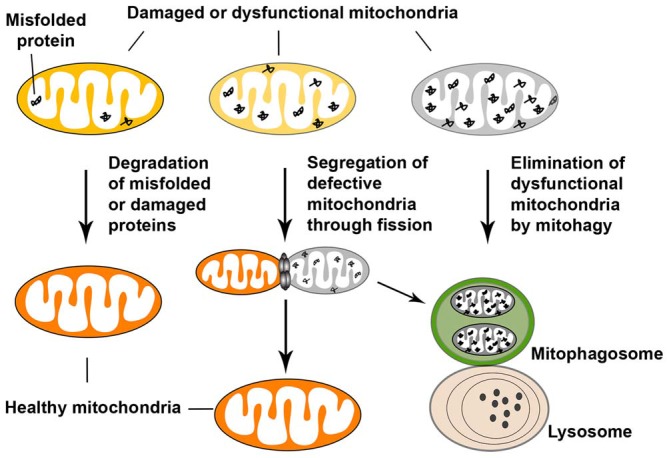
**Mitochondrial quality control.** Mitochondrial quality control occurs at multiple levels in order to limit mitochondrial damage and ensure mitochondrial integrity. At the molecular level of defense, degradation of misfolded or damaged mitochondrial proteins is supported by the proteolytic system. Molecular chaperones and ATP-dependent proteases in the matrix and inner membrane (IM) of mitochondria degrade damaged proteins, stabilize misfolded proteins (thus preventing their aggregation), and/or dissolve protein aggregates, and thereby promote proteolysis. In addition, the cytosolic ubiquitin-proteasome system can participate in the quality control of mitochondrial proteins. At the organellar level, mitochondrial fusion and fission provide additional protection against mitochondrial damage. Damaged mitochondria can be repaired by fusion with healthy mitochondria, which allows the contents of healthy and dysfunctional mitochondria to be mixed. Fission, on the other hand, segregates mitochondria that have become irreversibly damaged or are fusion-incompetent and results in their subsequent elimination by autophagy. If the two quality control pathways described above are ineffective, dysfunctional mitochondria are eliminated by autophagy. One type of cargo-specific autophagy is mitophagy, which selectively removes damaged mitochondria. Figure is modified from Sheng and Cai (2012).

Mitochondrial quality control involves surveillance and protective strategies at multiple levels in order to limit mitochondrial damage and ensure mitochondrial integrity. The quality control occurs at the molecular, organellar, and cellular levels. At the molecular level, a proteolytic system, involving molecular chaperones and ATP-dependent proteases in the matrix and the inner membrane (IM) of mitochondria, degrades damaged and misfolded proteins and/or dissolves protein aggregates for proteolysis (de Castro et al., 2010). The ubiquitin-proteasome system in the cytosolic additionally participates in the quality control of mitochondrial proteins (Tatsuta and Langer, 2008). At the organellar level, mitochondrial fusion and fission provide additional protection against mitochondrial damage. Dysfunctional mitochondria can be repaired by fusion with healthy mitochondria, which allows for mixture of the contents of healthy and defective mitochondria (Detmer and Chan, 2007; Chen and Chan, 2009; Westermann, 2010). In other cases, severely damaged mitochondria are segregated by fission, ultimately leading to their elimination within the autophagy-lysosomal system through mitophagy (Youle and Narendra, 2011). However, if the quality control pathways at the molecular and organellar levels are defective or if the levels of damage exceed the capacity of these two pathways, damaged mitochondria can rupture and release pro-apoptotic factors, leading to activation of apoptosis and cell death (Figure 4).

## Mitochondrial Dynamics and Mitophagy

Mitochondrial dynamics regulate mitochondrial elimination through the autophagy-lysosomal system. Mitochondrial elongation protects mitochondria from autophagy-mediated degradation (Gomes et al., 2011; Rambold et al., 2011). Parkin-mediated mitophagy can also be prevented by inhibition of Drp1-mediated fission prevents (Tanaka et al., 2010). Studies have indicated that mitochondrial fission yields uneven products, leaving one hyperpolarized mitochondrion and one depolarized daughter mitochondrion (Twig et al., 2008). Such depolarized mitochondria display reduced levels of OPA1 protein, causing them to become much less likely to fuse and to eventually undergo autophagocytosis. Additional evidence supports the notion that mitophagy relies on the loss of fusion and the presence of fission, as OPA1-overexpression, Fis1 RNAi, and Drp1 dominant-negative expression all reduce mitophagy levels (Narendra et al., 2008; Twig et al., 2008). Moreover, a study in yeast indicates that dynamin-related 1 (Dnm1) and other components of the mitochondrial fission machinery are dispensable for mitophagy (Mendl et al., 2011). However, it should be noted that mitochondrial fragmentation facilitates, but is not itself sufficient for, mitophagy. Reciprocally, mitophagy influences mitochondrial dynamics. Upon mitophagy induction, Parkin induces ubiquitination of Mfn1 and Mfn2, which leads to their degradation in a proteasome- and p97-dependent manner (Gegg et al., 2010; Poole et al., 2010; Tanaka et al., 2010; Ziviani et al., 2010; Chan et al., 2011). A recent study further showed that PINK1 phosphorylates Mfn2 and thereby promotes Parkin-mediated Mfn2 ubiquitination, which is required for the quality control of cardiac mitochondria (Chen and Dorn, 2013). Through this interplay between mitophagy and mitochondrial dynamics, Parkin-mediated mitophagy inhibits the fusion of defective mitochondria with healthy ones, thus promoting the segregation of dysfunctional mitochondria for degradation via the autophagy-lysosomal pathway.

## Mitochondrial Transport and Mitophagy

The mechanism coordinating mitochondrial motility and mitophagy represents an important emerging area. It has been suggested that a likely relationship between mitochondrial membrane potential and the direction of mitochondrial movement exists. One study showed that mitochondria with high membrane potentials exhibit anterograde transport towards distal processes, whereas depolarized mitochondria return to the soma following acute membrane potential dissipation (Miller and Sheetz, 2004). These findings suggest that damaged or defective mitochondria are delivered to the soma for repair and/or degradation, which is consistent with the observation that somatodendritic regions and the proximal axon are relatively enriched with mature lysosomes (Overly and Hollenbeck, 1996; Cai et al., 2010; Lee et al., 2011). Consistently, one recent study revealed that depolarized mitochondria in *PINK1* mutant *Drosophila* neurons showed reduced anterograde axonal transport and altered morphology in the soma, indicating that mitochondrial turnover might be restricted to the cell body *in vivo* in the intact nervous system (Devireddy et al., 2015). Another study reported that axonal mitochondria displayed reduced anterograde transport and relatively increased retrograde transport upon mitophagy induction, promoting the accumulation of Parkin-targeted mitochondria in the soma and proximal regions (Cai et al., 2012a,b). The altered motility may serve as a protective mechanism: healthy mitochondria remain in distal areas whereas aged and defective mitochondria are returned to the soma. This dynamic process allows neurons to efficiently remove defective mitochondria from distal regions, and then eliminate them within mature lysosomes in the soma.

Reduced anterograde transport of depolarized mitochondria is consistent with the results of many studies showing that Miro is degraded in response to mitophagy induction. During the early phase of mitophagy, the Parkin-activated ubiquitin-proteasome system was shown to mediate widespread degradation of Miro1 and Miro2 (Chan et al., 2011; Yoshii et al., 2011). In addition to binding to KIF5-Trak motor complex, Miro has been found to interact with Parkin and is ubiquitinated by Parkin following mitochondrial depolarization (Weihofen et al., 2009; Chan et al., 2011; Wang et al., 2011b; Liu et al., 2012b; Sarraf et al., 2013; Birsa et al., 2014). Degradation of Miro on the surfaces of defective mitochondria may not only suppress mitochondrial anterograde transport, but also favor their retrograde transport to the soma or immobilization at distal regions in preparation for mitophagy.

**Figure 5:**
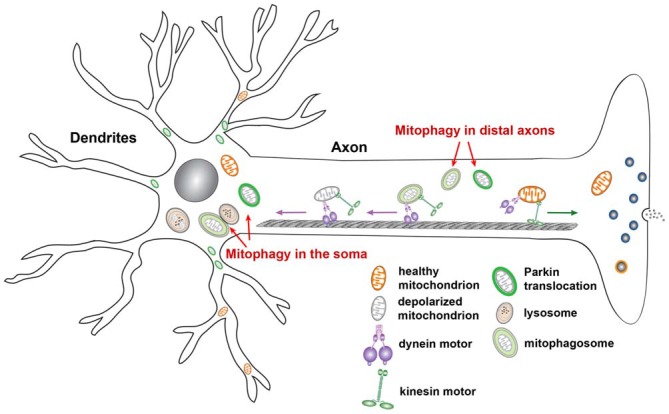
**Functional interplay of mitochondrial transport and mitophagy in neurons.** Upon mitochondrial membrane potential dissipation, Parkin-targeted mitochondria accumulate in the soma and proximal regions. Such compartmental restriction is attributed to altered motility of depolarized mitochondria, which exhibit reduced anterograde and relatively enhanced retrograde transport, thus reducing anterograde flux of damaged mitochondria into distal processes. This spatial process allows neurons to efficiently remove dysfunctional mitochondria from distal axons via the autophagy-lysosomal pathway in the soma, where mature lysosomes are mainly located. Damaged mitochondria at axonal terminals can also recruit Parkin for mitophagy once they are anchored by syntaphilin, or immobilized by turnover of the motor adaptor Miro on the mitochondrial surface. Autophagosomes containing engulfed mitochondria at axonal terminals are predominantly transported to the soma for maturation and for more efficient cargo degradation within acidic lysosomes. Figure is modified from Sheng (2014).

Once mitochondria are immobilized at distal axons by Miro degradation or by syntaphilin, the axonal mitochondrial-anchoring protein (Kang et al., 2008; Chen et al., 2009), damaged mitochondria may also recruit Parkin or be engulfed by autophagosomes (Cai et al., 2012a,b; Ashrafi et al., 2014). Recent studies indicate that autophagosomes, including those containing engulfed mitochondria, predominantly undergo retrograde transport from distal axons to the soma, which allows for autophagosome maturation and subsequent degradation within mature lysosomes in the proximal regions and soma of neurons (Maday and Holzbaur, 2014; Maday et al., 2012; Cheng et al., 2015). This functional interplay between mitochondrial motility and mitophagy is a particularly important mechanism by which the PINK1/Parkin pathway governs mitochondrial quality control for the proper removal of aged and defective mitochondria from distal axons and nerve terminals (Figure 5).

### Mitochondrial Abnormalities in Alzheimer’s Disease

In AD brains, mitochondria display multiple abnormalities, including impaired mitochondrial function, altered mitochondrial dynamics and transport, increased mtDNA mutations, defective mitochondrial enzyme activities, and abnormal expression of mitochondrial genes (Reddy et al., 2011). A growing body of evidence suggests that accumulation of amyloid precursor protein (APP) and amyloid β (Aβ) peptides plays a central role in mediating mitochondrial toxicity.

APP and Aβ have been found in the purified mitochondria from patient brains and AD mouse models (Anandatheerthavarada et al., 2003; Lustbader et al., 2004; Caspersen et al., 2005; Crouch et al., 2005; Devi et al., 2006; Manczak et al., 2006; Du et al., 2008; Yao et al., 2009). Aβ has been shown to interact with mitochondrial matrix proteins ABAD and cyclophilin D, which has been suggested to induce cytotoxic effects (Lustbader et al., 2004; Du et al., 2008). Aβ was also found to affect mitochondrial fusion and fission (Wang et al., 2008b; Manczak et al., 2011; Manczak and Reddy, 2012b), alter mitochondrial motility (Rui et al., 2006; Du et al., 2010; Calkins et al., 2011), disrupt function of the electron transfer chain, increase ROS production (Keller et al., 1997; Abramov et al., 2004; Manczak et al., 2006), and impair mitochondrial function (Mattson et al., 1998; Lustbader et al., 2004; Du et al., 2008). Neurons derived from mutant APP transgenic (Tg) mouse models display altered mitochondrial dynamics, impaired trafficking, and reduced biogenesis (Calkins et al., 2011; Trushina et al., 2012). Abnormal accumulation of Aβ within synaptic mitochondria was proposed to contribute to early deficits in synaptic function in AD (Du et al., 2010). Moreover, the degree of cognitive impairment in AD brains has been linked to the extent of mitochondrial dysfunction and mitochondrial Aβ accumulation (Dragicevic et al., 2010).

While it remains to be experimentally proven whether intramitochondrial production of Aβ occurs (Pinho et al., 2014), studies have shown possible routes for Aβ entry into mitochondria—via mitochondrial-associated endoplasmic reticulum membrane (MAM; Pinho et al., 2014) or via the translocase of the outer mitochondrial membrane (TOM) complex (Hansson Petersen et al., 2008). Extracellular Aβ was also shown to be internalized and taken up by mitochondria (Hansson Petersen et al., 2008; Hedskog et al., 2013). One recent study reported that reduction in mitochondrial membrane potential and the emergence of dystrophic and fragmented mitochondria were limited to the vicinity of Aβ plaques in a live AD mouse model, suggesting that Aβ plaques likely serve as focal sources that promote mitochondrial Aβ accumulation and thus Aβ-mediated toxicity (Xie et al., 2013). Altogether, these pieces of evidences indicate that the accumulation of APP and Aβ in the mitochondrial compartment likely has a causative role in altering mitochondrial dynamics and transport, thereby leading to mitochondrial dysfunction.

### Abnormal Mitochondrial Dynamics in Alzheimer’s Disease

Mitochondrial fragmentation and reduced mitochondrial density in neuronal processes have been consistently observed in neurons exposed to Aβ-derived diffusible ligands (ADDLs) or oligomeric Aβ, as well as in primary neurons cultured from AβPP mice (Wang et al., 2009a; Du et al., 2010; Calkins et al., 2011). One recent study provided *in vivo* evidence of the emergence of dystrophic and fragmented mitochondria and a reduction in the total number of mitochondria near amyloid plaques in living AD mouse brains (Xie et al., 2013). In another study, neuronal cells incubated with conditional medium from cells stably expressing mutant forms of APP showed an increase in mitochondrial fission, caused by elevated levels of S-nitrosylated Drp1 (SNO-Drp1; Cho et al., 2009). Enhanced dimerization of SNO-Drp1, which was found in AD patient brains and mouse models, likely contributes to increased fission activity. These observations suggest that Aβ-mediated cytotoxic effects lead to enhanced Drp1 activity through the generation of nitric oxide. One study found the contrary result of reduced levels of Drp1 in fibroblasts from sporadic AD patients and AD patient brains (Wang et al., 2008a, 2009a). The same group provided further evidence that overexpression of APP in M17 neuroblastoma cells results in predominant mitochondrial fragmentation and decreased levels of Drp1 and OPA1, while overexpression of Drp1 or OPA1 could partially rescue some of these defects (Wang et al., 2008b). However, more recent studies found increased levels of Drp1 and Fis1 and reduced expression of Mfn1, Mfn2 and OPA1 in AD patient brains. Moreover, increased Aβ production and the interactions of Drp1 with Aβ and phosphorylated tau lead to abnormal mitochondrial fragmentation. These abnormal interactions are increased as AD progresses (Manczak et al., 2011; Reddy et al., 2011; Manczak and Reddy, 2012a; Kandimalla and Hemachandra Reddy, 2015). Therefore, impaired balance in mitochondrial fusion and fission in AD neurons likely interferes with mitochondrial motility and mitophagy, thereby compromising mitochondrial quality control.

## Alterations in Mitochondrial Transport in Alzheimer’S Disease

Several lines of evidence support the hypothesis that impaired axonal transport plays an important role in the pathogenesis of AD (Stokin and Goldstein, 2006; Wang et al., 2009b). Axonal degeneration in patients with AD is characterized by swollen regions where abnormal amounts of organelles (including mitochondria) accumulate (Stokin et al., 2005). It was reported that presenilin 1 (PS1) mutations impair kineisin-1 based axonal transport by increased GSK3β activity and thereby enhancing the phosphorylation of kinesin-1 light chains (KLC). This defect leads to a reduction in the densities of APP, synaptic vesicles and mitochondria in the neuronal processes of hippocampal neurons and sciatic nerves from mutant PS1 knock-in mice (Pigino et al., 2003). Consistent with this finding, a study utilizing real-time analysis of vesicle motility demonstrated that isolated axoplasms perfused with soluble intracellular oligomeric Aβ exhibited inhibition of bidirectional axonal transport as a result of increased phosphorylation of KLC and subsequent release of kinesin from its cargoes (Pigino et al., 2009). These observations suggest that defects in axonal transport may compromise neuronal function by interfering with both trafficking and distribution densities of important cargoes, including mitochondria, in distal axons and at nerve terminals.

Exposure of cultured neurons to Aβ or ADDLs consistently results in decreased mitochondrial motility and reduced mitochondrial density in axons (Rui et al., 2006; Du et al., 2010; Vossel et al., 2010; Wang et al., 2010). One study showed than overexpression of Aβ in *Drosophila* slowed down bidirectional transport of axonal mitochondria and depleted presynaptic mitochondria, leading to presynaptic dysfunction (Zhao et al., 2010). Primary neurons derived from AβPP Tg mice also exhibited impaired anterograde transport of axonal mitochondria, mitochondrial dysfunction, and synaptic deficiency, which could be attributed to the accumulation of oligomeric Aβ in mitochondria (Calkins et al., 2011). Mitophagy induction is associated with alterations in mitochondrial motility. As a result of Parkin-mediated degradation of Miro, mitophagy is accompanied by reduced anterograde mitochondrial transport (Chan et al., 2011; Wang et al., 2011b; Cai et al., 2012a,b; Liu et al., 2012b; Bingol et al., 2014; Birsa et al., 2014). Our recent study showed that Parkin-mediated mitophagy is robustly induced in AD neurons of mouse models and patient brains. Consequently, these neurons display reduced anterograde transport of axonal mitochondria (Ye et al., 2015). Application of a mitochondria-targeted antioxidant was shown to reverse these defects and restore mitochondrial motility in AD neurons (Calkins et al., 2011), likely by attenuating mitophagy and Miro degradation. These studies show that abnormal mitochondrial motility in AD neurons may be attributed to the induction of mitophagy. Together, defective mitochondrial transport and reduced mitochondrial density in distal axons and at synaptic terminals may cause local energy depletion and toxic changes in Ca^2+^ buffering, thus triggering synaptic dysfunction and loss in the pathogenesis of AD.

## Abnormal Autophagy and Mitophagy in Alzheimer’S Disease

Mounting evidence has implicated defective autophagy in the pathogenesis of AD (Nixon, 2007, 2013; Nixon and Yang, 2011). This is evidenced in AD brains by the massive accumulation of AVs in the soma and within large swellings along dystrophic and degenerating neurites. These serve as major reservoirs of intracellular Aβ and create conditions favorable for Aβ accumulation (Nixon et al., 2005; Yu et al., 2005; Nixon, 2007). However, it is largely unknown what cellular basis contributes to altered autophagy in affected AD neurons. Previous studies reported prominent autophagic accumulation of mitochondria in AD patient brains, suggesting increased mitochondrial turnover by autophagy (Hirai et al., 2001; Moreira et al., 2007a,b). These findings suggest that altered autophagy or mitophagy contributes to mitochondrial defects in AD brains. Despite enhanced autophagy induction, AD brains still exhibit aberrant accumulation of ultrastructurally altered mitochondria with reduced size and broken internal membrane cristae (Hirai et al., 2001; Baloyannis, 2006; Moreira et al., 2007a,b; Trushina et al., 2012).

Our recent study showed that enhanced mitophagy induction contributes to increased autophagic flux in AD (Ye et al., 2015). In both mutant hAPP neurons and AD patient brains, robust induction of Parkin-mediated mitophagy was observed. In the absence of mitochondrial membrane potential dissipation reagents, hAPP neurons exhibit increased recruitment of cytosolic Parkin to depolarized mitochondria. Under AD-linked pathophysiological conditions, Parkin translocation predominantly occurs in the somatodendritic regions, coupled with reduced anterograde and increased retrograde transport of axonal mitochondria. As the disease progresses, this enhancement of mitophagy in AD patient brains is accompanied by a depletion of cytosolic Parkin. Consistent with this, overexpression of Parkin in an AD mouse model was shown to enhance autophagic clearance of defective mitochondria and prevent mitochondrial dysfunction (Khandelwal et al., 2011; Martín-Maestro et al., 2015). Thus, these data indicate that aberrant accumulation of defective mitochondria in AD-affected neurons is likely caused by inadequate mitophagy capabilities for eliminating the increased numbers of damaged mitochondria.

Deficits in lysosomal degradation capacity have been implicated in AD as well, as it leads to the accumulation of proteolytic substrates in affected neurons (Boland et al., 2008; Lee et al., 2010, 2011; Yang et al., 2011). One recent study provided evidence showing that endolysosomal deficits augment mitochondrial pathology in the motor neurons of an ALS mouse model (Xie et al., 2015). Defective lysosomal proteolysis likely impairs turnover of mitophagosomes, thereby contributing to their aberrant accumulation in AD brains. While enhanced mitophagy induction contributes to increased autophagic flux, autophagy failure as a result of lysosomal deficits leads to inadequate mitophagic clearance and therefore mitochondrial pathology observed in AD.

## Abnormal Mitochondrial Quality Control in Alzheimer’S Disease

**Figure 6:**
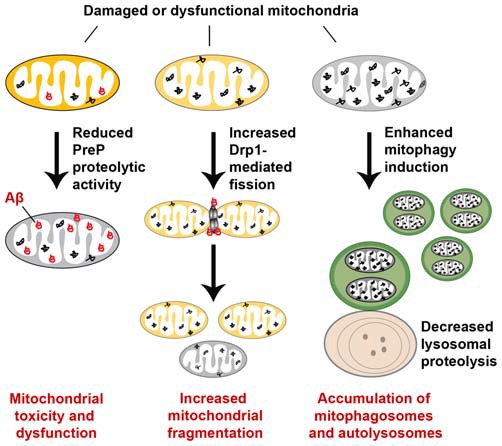
**Abnormal mitochondrial quality control in Alzheimer’s disease (AD).** Mitochondrial quality control is impaired at multiple levels in AD. At the molecular level, the mitochondrial presequence protease (PreP) degrades Aβ within mitochondria and thus reduces its toxic effects on mitochondria. However, the proteolytic activity of PreP is impaired as a result of increased reactive oxygen species (ROS) production in AD, thereby promoting accumulation of Aβ and Aβ-mediated mitochondrial toxicity. A decrease in proteasome activity could contribute to altered quality control of other presequence peptides and mitochondrial proteins. Mitochondrial quality control at the organellar level is also disrupted in AD. Elevated Drp1 levels or activity and reduced levels of Mfn1 and Mfn2 result in mitochondrial fragmentation and reduced fusion, thus preventing damaged mitochondria from being repaired through the fusion-mediated mixture of contents with healthy mitochondria. Enhanced mitophagy induction and defective lysosomal proteolysis result in aberrant accumulation of damaged mitochondria within mitophagosomes and autolysosomes, which also contribute to impaired mitochondrial quality control in AD. Moreover, defects in axonal transport and mitochondrial motility compromise mitochondrial quality control by hindering dysfunctional mitochondria from being returned to the soma for lysosomal degradation.

Mitochondrial quality control, essential for limiting mitochondrial damage and maintaining mitochondrial integrity and function, occurs at the molecular, organellar, and cellular levels. Defects in each level interfere with the efficiency of quality control, thus leading to mitochondrial dysfunction or abnormal accumulation of damaged mitochondria. AD neurons have been shown to exhibit mitochondrial abnormalities including an imbalance in mitochondrial dynamics, impaired axonal transport, and inadequate mitophagy capacity, all of which may disrupt efficient elimination of dysfunctional mitochondria. Thus, mitochondrial pathology in AD can likely be attributed to impaired quality control (Figure 6).

Mitochondrial quality control at the molecular level is altered in AD. The mitochondrial presequence protease (PreP) was shown to degrade Aβ within mitochondria and thus reduce its toxic effects on mitochondrial function (Falkevall et al., 2006; Alikhani et al., 2011). However, in AD patient brains and mouse models, studies showed that increased ROS production impairs the proteolytic activity of PreP, thereby promoting accumulation of Aβ and Aβ-mediated mitochondrial toxicity. A decrease in proteasome activity could also compromise the quality control of other presequence peptides and mitochondrial proteins (Gregori et al., 1995; Keller et al., 2000; Tseng et al., 2008). As a result, the potential toxic effect caused by accumulation of damaged presequence peptides and mitochondrial proteins could further exacerbate mitochondrial dysfunction in AD (Teixeira and Glaser, 2013).

Mitochondrial quality control at the organellar level—involving mitochondrial dynamics, motility, and mitophagy—is also disrupted in AD. The important process of mitochondrial fusion typically allows damaged mitochondria to be repaired through fusion with healthy mitochondria, while mitochondrial fission segregates severely and irreversibly damaged mitochondria for their clearance via the autophagy-lysosomal pathway. AD brains exhibit an imbalance in mitochondrial fusion and fission, showing mitochondrial fragmentation and reduced fusion (Cho et al., 2009; Du et al., 2010; Calkins et al., 2011; Manczak et al., 2011; Manczak and Reddy, 2012a). Mitochondrial fragmentation is likely attributed to elevated Drp1 levels or activity and reduced levels of Mfn1 and Mfn2. Its enhanced occurrence promotes mitochondrial elimination via mitophagy, causing this process to become dominant. Meanwhile, suppressed fusion may prevent damaged mitochondria from being repaired through the fusion-mediated mixture of contents with healthy mitochondria. Thus, altered mitochondrial dynamics in AD neurons will interfere with mitochondrial quality control.

Retrograde transport of axonal mitochondria facilitates removal of aged and defective mitochondria from distal axons and nerve terminals, since mature lysosomes are mainly located in the soma (Overly and Hollenbeck, 1996; Cai et al., 2010, 2012a,b; Lee et al., 2011; Ye et al., 2015). Decreased mitochondrial motility has been observed in neurons exposed to Aβ or ADDLs, or in neurons cultured from AβPP Tg mice, and in *Drosophila* following Aβ overexpression (Rui et al., 2006; Du et al., 2010; Vossel et al., 2010; Wang et al., 2010; Zhao et al., 2010; Calkins et al., 2011). Such defects in axonal transport and mitochondrial motility compromise mitochondrial quality control by hindering dysfunctional mitochondria from being returned to the soma for lysosomal degradation. As a result, defective mitochondria are abnormally accumulated in the distal regions of AD neurons.

The key pathway of mitochondrial quality control, mitophagy, is also altered in AD. Among the various types of mitophagy, the PINK1/Parkin-mediated mitophagy in AD has been the focus of most current studies. Earlier studies revealed autophagic accumulation of mitochondria in AD patient brains (Hirai et al., 2001; Moreira et al., 2007a,b), suggesting enhanced turnover of damaged mitochondria via the autophagy-lysosomal pathway. We further showed that Parkin-mediated mitophagy, is robustly induced in AD patient brains and mouse models (Ye et al., 2015). However, AD brains exhibit depletion of cytosolic Parkin throughout the disease’s progression, which is consistent with a recent study’s findings that Parkin is diminished in the fibroblasts and brain biopsies of AD patients, leading to PINK1 accumulation (Martín-Maestro et al., 2015). Overexpression of Parkin in these cells rescues mitophagy failure by inducing recovery of mitochondrial membrane potential, as well as reducing PINK1 levels and the accumulation of abnormal mitochondria. These observations indicate that defective mitophagy in AD causes the aberrant accumulation of dysfunctional mitochondria. In addition, degradation of mitophagosomes relies on proper lysosomal function. Deficits in lysosomal proteolysis of autophagic substrates may further compromise mitophagic elimination of defective mitochondria, thus exacerbating AD pathology. Given that endolysosomal deficits were shown to augment mitochondrial pathology in ALS (Xie et al., 2015), it is possible that similar deficits may contribute to mitochondrial dysfunction in AD. Moreover, altered mitochondrial dynamics and reduced mitochondrial motility may interfere with mitophagy in AD, although no studies have yet confirmed this relationship. Therefore, the molecular interplay between abnormal mitochondrial fission, fusion, transport, and defective mitophagy must be further investigated to advance our understanding of mitochondrial quality control in AD. This represents an important research field, as a variety of neurodegenerative diseases are associated with mitochondrial dysfunction.

Altogether, alterations in mitochondrial fusion and fission, transport, mitophagy, and lysosomal proteolysis lead to disrupted mitochondrial quality control, thus augmenting mitochondrial pathology in AD brains.

## Tau-Mediated Mitochondrial Defects

Neurofibrillary tangle formation, composed mainly of hypophosphorylated tau, is also a pathological hallmark of AD. There is little information regarding tau-mediated regulation of mitochondria, and it remains largely unknown whether tauopathy contributes to mitochondrial dysfunction in AD. It has been shown that expression of human tau results in elongation of mitochondria in both *Drosophila* and mouse neurons by blocking recruitment of Drp1 to mitochondria, which is accompanied by mitochondrial dysfunction and cell death (DuBoff et al., 2012). As these phenotypes can be rescued by genetically restoring the proper balance of mitochondrial fusion and fission, it suggests that tau plays a role in regulating mitochondrial dynamics.

As for disease-related pathogenic tau mutants, overexpression of mutant tau—causing frontotemporal dementia with Parkinsonism linked to chromosome 17 (FTDP-17)—decreased fusion and fission rates due to reduced levels of OPA-1 and Drp-1. In contrast, overexpressing wild-type tau was shown to have protective effects on both mitochondrial dynamics and function, including the enhancement of complex I activity (Schulz et al., 2012). One study reported that hypophosphorylated tau induced mitochondrial fission and excessive mitochondrial fragmentation in postmortem brain tissues from patients with AD and mouse models by directly interacting with Drp1 (Manczak and Reddy, 2012a). In addition to its effects on fission, phosphorylated tau was shown to interact with VDAC in AD brains, leading to mitochondrial dysfunction likely by blocking mitochondrial pores (Manczak and Reddy, 2012b). These studies indicate that pathogenic forms of tau affect mitochondrial function directly through interaction with VDAC or indirectly through interference with Drp1-mediated fission.

Tau is a microtubule-associated protein (MAP) responsible for stabilizing axonal MTs. It has also been shown to regulate axonal transport of membranous organelles, including mitochondria (Stamer et al., 2002). Overexpressing tau selectively inhibits kinesin-driven anterograde mitochondrial transport in N2a and NB2a/d1 neuroblastoma cell lines, primary cortical neurons, and retinal ganglion neurons (Stamer et al., 2002; Dubey et al., 2008; Stoothoff et al., 2009). These studies suggest that tau preferentially competes with kinesin motors for binding to MTs. One study showed that the binding of tau to MTs can reverse the direction in which the dynein motor moves, whereas the same process tended to cause kinesin to detach from the MTs (Dixit et al., 2008). Interestingly, complete or partial loss of tau expression in mutant neurons prevents Aβ-mediated defects in axonal transport of mitochondria (Vossel et al., 2010). This suggests that the ability of Aβ to inhibit mitochondrial motility is dependent on tau expression levels. Thus, perturbing tau expression and distribution in axons would impair axonal transport and lead to neurodegeneration.

While mutant tau expression results in mitochondrial dysfunction, deregulation of mitochondrial dynamics, and impaired mitochondrial transport, one recent study reported that pathogenic tau truncation might also contribute to abnormal mitophagy in AD. A 20–22 kDa NH2-tau fragment detectable in cellular and animal AD models and human AD subjects was shown to be stably associated with Parkin and UCHL-1. As a result, it led to aberrant recruitment of Parkin and UCHL-1 to mitochondria and excessive Parkin-dependent mitochondrial turnover (Corsetti et al., 2015). Suppression of this improper mitophagy restores mitochondrial content at synapses and results in partial, but significant, protection against the NH2-htau-induced neuronal death. Altogether, the pathogenic forms of tau-mediated mitochondrial alterations—including impaired balance of mitochondrial fusion and fission, reduced mitochondrial motility, and excessive mitophagy—ultimately interfere with mitochondrial quality control, thus contributing to mitochondrial dysfunction in AD.

## Concluding Remarks

Mitochondrial dysfunction is a prominent feature in AD, but it remains unclear whether the cellular mechanisms maintaining mitochondrial quality are defective and further augments mitochondrial pathology. Mitochondrial quality control involves multiple levels of surveillance and protective strategies to limit mitochondrial damage and efficiently eliminate defective mitochondria for the maintenance of mitochondrial homeostasis. Mitochondrial fusion and fission control mitochondrial shape and function, while mitochondrial transport plays a critical role in regulating mitochondrial distribution and removing aged and damaged mitochondrial from distal axons and synapses for lysosomal degradation in the soma. These key features of mitochondria operate strongly in conjunction with mitophagy as the key pathways of mitochondrial quality control. Studies reviewed here suggest that a complex network of dynamic and reciprocal interactions among mitochondrial fusion, fission, transport, and mitophagy governs mitochondrial integrity and function. Accumulating evidence notes that perturbed mitochondrial dynamics and abnormal mitophagy exist in AD brains (Chen and Chan, 2009; Sheng and Cai, 2012), which may directly or indirectly interfere with the quality control of mitochondria. Further investigation into these mechanisms would advance our understanding of mitochondrial dysfunction in AD. Given that manipulation of genes controlling mitophagy can ameliorate some phenotypes (Khandelwal et al., 2011; Martín-Maestro et al., 2015), there is compelling reason to hope that efforts to artificially manipulate mitochondrial dynamics, motility, and mitophagy will enhance mitochondrial surveillance mechanisms and attenuate the neuropathology of AD, ultimately yielding new therapeutic approaches for the devastating disease.

## Author Contributions

QC conceived ideas and wrote the manuscript. PT contributed to some sections related with AD.

## Conflict of Interest Statement

The authors declare that the research was conducted in the absence of any commercial or financial relationships that could be construed as a potential conflict of interest.
